# Combined Therapy of Diabetic Peripheral Neuropathy with Breviscapine and Mecobalamin: A Systematic Review and a Meta-Analysis of Chinese Studies

**DOI:** 10.1155/2015/680756

**Published:** 2015-03-19

**Authors:** Chanjiao Zheng, Weilin Ou, Huanyu Shen, Zhiheng Zhou, Jiaji Wang

**Affiliations:** School of Public Health, Guangzhou Medical University, Guangzhou 510182, China

## Abstract

*Objective*. A meta-analysis on combined therapy of diabetic peripheral neuropathy (DPN) with breviscapine and mecobalamin was performed to evaluate the efficacy of this therapy. *Methods*. Six English databases (Medline, Cochrane Library, PubMed, EMBASE, Web of Science, and CINAHL) and four Chinese databases (China National Knowledge Infrastructure, VIP Journals Database, CBM, and Wanfang database) were searched for studies on the clinical trials in which DPN was treated with breviscapine and mecobalamin, and RevMan 5.1 package was employed for analyzing pooled trials and publication bias. *Results*. A total of 17 articles including 1398 DPN patients were identified. Homogeneity was observed among different studies (*P* = 0.74). The efficacy of combined therapy with breviscapine and mecobalamin was significantly better than that in control group [*P* < 0.0001 (OR = 5.01, 95% CI: 3.70–6.78)]. *Conclusion*. Available findings suggest that the therapeutic efficacy of breviscapine combining mecobalamin is superior to mecobalamin alone, and this strategy is required to be popularized in clinical practice.

## 1. Introduction

Diabetic peripheral neuropathy (DPN) is a diabetes mellitus (DM) induced disorder of the peripheral nervous system [1] and is characterized by the pain and loss of sensation due to symmetrical degeneration of distal peripheral nerves. The symptoms will deteriorate with the progression, which may result in diabetic ulcers or even nontraumatic amputation. Statistics revealed that the incidence of DPN was as high as 30%, 60%, and 90% at 5, 10, and 20 years after diagnosis of DM, and foot injury had occurred in 50% of DPN patients when they were asymptomatic [2]. The incidence of neuropathy is now estimated to be about 8% in new cases of DM, and neuropathy will be a lifelong disease in more than 50% of DM patients, which is about 4 times the figure (12.3%) in DM patients in 2001 [1, 3, 4]. Thus, DPN has been an important economic burden of the medical system [5] and significantly influenced the quality of life of DM patients. The pathogenesis of DPN is complicated and still poorly understood [6, 7]. Studies have found that DPN was closely associated with metabolic disorder, vascular diseases, and oxidative stress [8–10]. Currently, pharmacotherapy of DPN is mainly to relieve pain with tricyclic antidepressants, anticonvulsants (gabapentin, phenytoin, lamotrigine, opioids, and tramadol), focal analgesics (capsaicin), and nonsteroidal anti-inflammatory drugs [11]. In addition, studies also revealed that vitamin B12 was also beneficial for the improvement of symptoms of DPN patients [12].

Breviscapine is an active ingredient of flavonoids extracted from dried* Erigeron breviscapus* (Vant.) Hand. Mazz. [13, 14]. In clinical practice, breviscapine tablets and breviscapine injections are mainly used in the therapy of various diseases. There were evidences showing that breviscapine was able to dilate blood vessels, reduce vascular resistance, increase blood flow, improve microcirculation, inhibit angiogenesis, and suppress aggregation of platelets [14–18]. Breviscapine may act as an antioxidant [19–21] and has been used in the therapy of DPN in China. Mecobalamin is an endogenous coenzyme B12 and can be used alone or in combination with other drugs. It is also widely used in the therapy of DPN [22–25]. Previous Chinese studies revealed that combined therapy of breviscapine and mecobalamin had better efficacy and safety for DPN when compared with mecobalamin alone [26, 27]. Although several randomized controlled trials have been conducted in the combined therapy of DPN with breviscapine and mecobalamin, the sample size was small and the test potency was low, resulting in low reliability of these studies. In this meta-analysis, studies on the combined therapy of DPN with breviscapine and mecobalamin were selected for pooled analysis, aiming to evaluate the therapeutic efficacy of this strategy in DPN patients.

## 2. Materials and Methods

### 2.1. Literature Searching and Data Extraction

A comprehensive literature search was performed for the randomized, controlled studies on the combined therapy of DPN with breviscapine and mecobalamin published before September 2014 by using Medline database (1989 to September 2014), Cochrane Library (1993 to September 2014), PubMed (1966 to September 2014), EMBASE (1980 to September 2014), Web of Science (1945 to September 2014), CINAHL (1982 to September 2014), CNKI database (1979 to September 2014), Chinese Biomedical Literature database (1990 to September 2014), Wanfang database (1982 to September 2014), and VIP database (1989 to September 2014). The following terms were used: (DPN OR diabetic peripheral neuropathy OR diabetic neuropathy) AND (breviscapine OR erigeron breviscapus). Searching was done by two authors (Weilin Ou and Huanyu Shen) independently.

### 2.2. Inclusion and Exclusion Criteria

#### 2.2.1. Inclusion Criteria Included

(1) The internationally accepted diagnostic criteria were used in these studies: DM was diagnosed according to the WHO criteria for diabetes mellitus in 1999 [27], patients had related symptoms of motor and sensory nerves, and other causes of peripheral neuropathy were excluded (such as hypothyroidism, genetics, alcoholism, and drugs); (2) studies were clinically randomized controlled trials; (3) patients were treated with mecobalamin alone in control group; patients were treated with mecobalamin and breviscapine in intervention group; (4) studies investigated the therapeutic efficacy or the changes in the conduction velocity of motor or sensory nerves.

#### 2.2.2. Exclusion Criteria Included

(1) There was no control group, or patients in control group were not treated with mecobalamin; (2) other parameters were used for evaluation of therapeutic efficacy; (3) studies were descriptive trials; (4) mecobalamin or breviscapine in combination with other drugs was used to treat DPN.

### 2.3. Data Extraction

Relevant data was systematically collected from each included study by two authors (Weilin Ou and Huanyu Shen) using a standardized form. The following information was extracted: number of patients in different groups, age, gender, course of DM, course of DPN, study duration, daily dose of breviscapine, and endpoints. This information was collected independently by two authors and inconsistence was resolved after consultation with a third investigator (Chanjiao Zheng).

### 2.4. Outcomes

There is no unified curative effect evaluation standard. So evaluation index was chosen according to the selected trials to evaluate curative effect. The authors in the 17 articles were observing two groups of patients before and after treatment about the change of subjective symptom and lower limb nerve reflex and using the electromyography tested the motor nerve conduction velocity (MNCV) and sensory nerve conduction velocity (SNCV) of total nerve and median nerve before and after treatments. Therapeutic effect criteria include the following [28]: (1) excellence: self-conscious symptoms were markedly improved, obvious tendon reflexes improved or recovered, and the MNCV/SNCV increased by more than 5 m/s or back to normal; (2) effectiveness: self-conscious symptoms were improved, tendon reflexes improved or recovered, and the MNCV/SNCV increased by less than 5 m/s or increased slightly; (3) invalidism: self-conscious symptoms were not improved and tendon reflexes and MCV/SC did not change. Total efficiency was equal to the excellence and effectiveness. The primary outcomes included therapeutic efficacy and absolute values of or changes in median MNCV, peroneal MNCV, median SNCV, and peroneal SNCV. The secondary outcomes included the improvement of clinical symptoms (overall effectiveness) and side effects. The effectiveness was defined as the improvement of clinical symptoms, tendon reflexes, and nerve conduction velocity.

### 2.5. Quality Assessment

The quality assessment of studies was done with the Cochrane Collaboration's tool (The Cochrane Library) which contained random sequence generation, allocation concealment, blinding of participants and personnel, blinding of outcome assessment, incomplete outcome data, selective outcome reporting, and other potential sources of bias. The outcome of each item was classified as low risk of bias, unclear risk of bias, and high risk of bias. The quality assessment was performed independently by two investigators (Weilin Ou and Chanjiao Zheng) and inconsistence was resolved after consultation with a third investigator (Jiaji Wang).

### 2.6. Data Synthesis and Analysis

Review Manager 5.1 software was used for the analysis of data. The odds ratio (OR) and 95% confidence interval (CI) were calculated for dichotomic data, and quantitative data were expressed as weighted mean difference (WMD) and 95% CI. Heterogeneity analysis was done with *q* test. *P* > 0.1 and *I* ^2^ < 50% suggested homogeneity among studies. For data without significant heterogeneity, fixed effects model was employed for pooled analysis. When significant heterogeneity (*P* ≤ 0.1 and *I* ^2^ ≥ 50%) was present, random effects model was employed for pooled analysis. The significance of pooled data was further tested, and a value of *P* < 0.05 was considered statistically significant. When enough studies were included, funnel plot was delineated and the publication bias was evaluated.

## 3. Results

### 3.1. Study Selection

**Figure 1:**
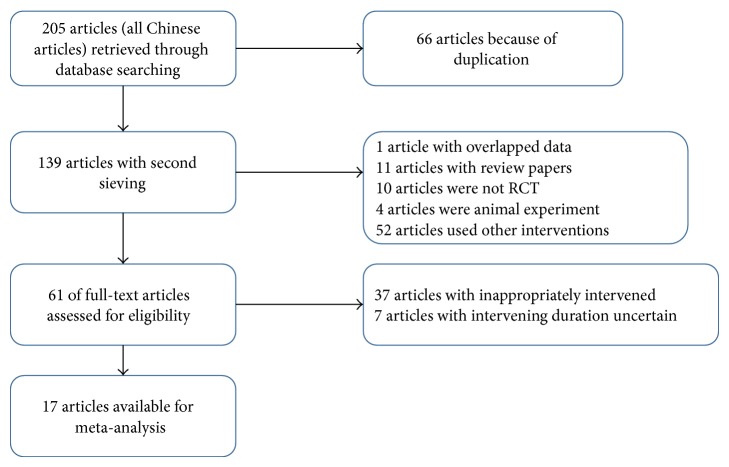
Flowchart of study selection.

A total of 205 literatures were identified after searching. Among them, 66 were identical in different databases, 1 was duplicated, 11 were retrospective reviews, 10 were noncontrolled studies, 4 were studies on animals, and other 52 literatures were excluded due to other reasons. The remaining 61 literatures were included for the evaluation of full text. Of these 61 literatures, the interventions were inappropriate in 37 and the duration of therapy was indefinite in 7 literatures. Thus, 17 articles were finally eligible for meta-analysis [25, 26, 28–42]. The flowchart of study selection is shown in Figure 1.

### 3.2. Evaluation of Quality of Included Studies

**Figure 2:**
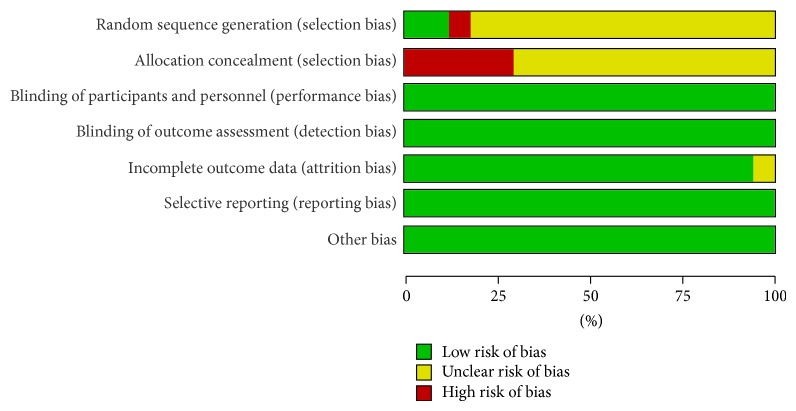
Risk of bias across studies assessed using the Cochrane risk of bias tool.

**Figure 3:**
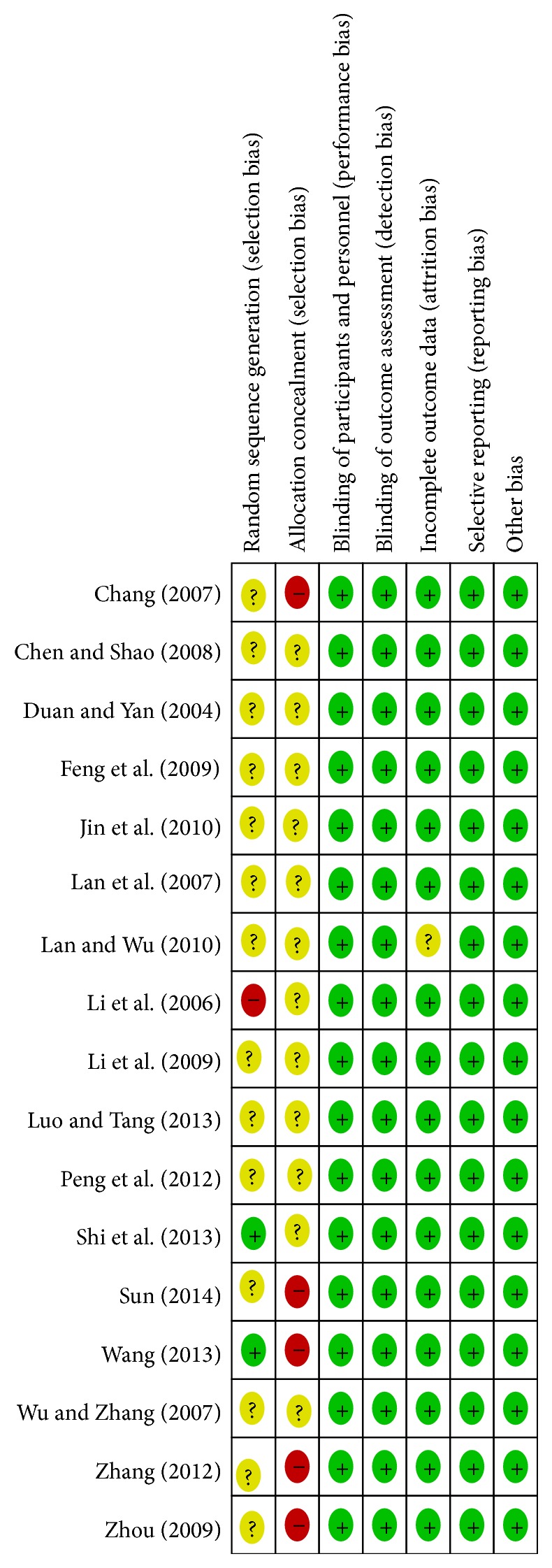
Risk of bias in individual studies using Cochrane risk of bias.

Among 17 studies, there were 1398 patients including 718 treated with breviscapine and mecobalamin and 680 treated with mecobalamin alone. In these studies, patients received diet control, excising, and glucose-lowering therapy before interventions. In most of the studies, patients were treated continuously for 2–6 weeks, except for intervals of 2-3 days in 2 studies [32, 40] and an interval of 2 weeks in 1 study [25]. The detailed information of included studies is shown in Table 1. Among 17 studies, randomized grouping was addressed in 1 study [39], random number method was used in 1 study [25], and only randomization was addressed in remaining studies but the specific method for randomization was not described. Of these studies, only 1 was classified as high risk of bias, and the quality of included studies is shown in Figures 2 and 3.

### 3.3. Meta-Analysis

#### 3.3.1. Overall Effectiveness on the Basis of Improvement of Clinical Symptoms and Signs

**Figure 4:**
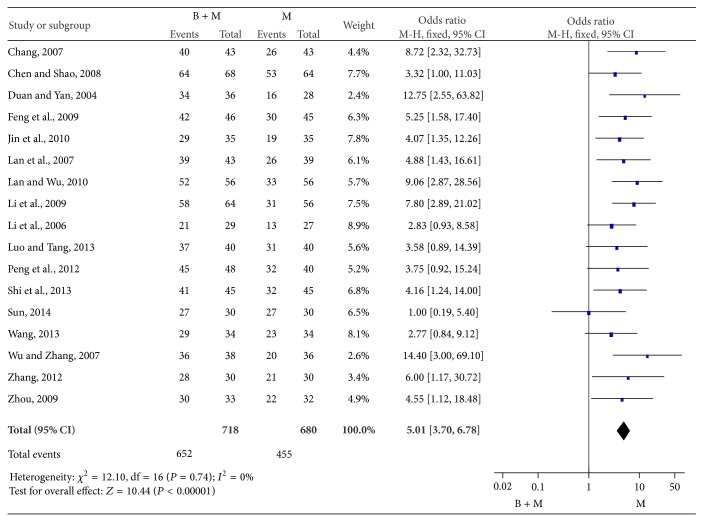
Overall effectiveness of therapy with breviscapine and mecobalamin versus mecobalamin.

A total of 17 studies were conducted to evaluate the therapeutic efficacy of breviscapine and mecobalamin as compared to that of mecobalamin alone. There were 718 patients treated with breviscapine and mecobalamin and 680 patients treated with mecobalamin alone in meta-analysis. There was no heterogeneity among groups (*P* = 0.74, *I* ^2^ = 0%), and fixed effects model was employed for pooled analysis which showed that OR was 5.01, 95% CI was 3.70–6.78, *Z* was 10.44 (*P* < 0.0001), and the diamond on the right side of the vertical line was complete, suggesting that the overall effectiveness of breviscapine and mecobalamin was significantly superior to that of mecobalamin alone (Figure 4).

#### 3.3.2. Meta-Analysis of Nerve Conduction Velocity after Therapy

**Figure 5:**
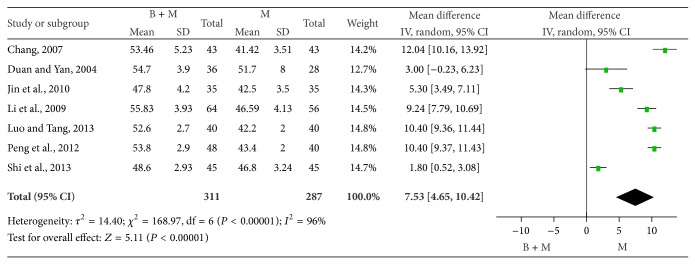
Median MNCV of two groups after therapy.

*(1) Median MNCV.* The median MNCV was compared between groups in 7 studies [25, 28, 29, 31, 33, 36, 37]. There were 311 patients treated with breviscapine and mecobalamin and 287 patients treated with mecobalamin alone. There was significant heterogeneity among groups (*P* < 0.0001, *I* ^2^ = 96%), and random effects model was employed for pooled analysis. The pooled WMD was 7.53, 95% CI was 4.65–10.42, *Z* was 5.11 (*P* < 0.0001), and the diamond on the right side of the vertical line was complete, suggesting that the median MNCV of patients treated with breviscapine and mecobalamin was significantly higher than that of patients treated with mecobalamin alone (Figure 5). 

**Figure 6:**
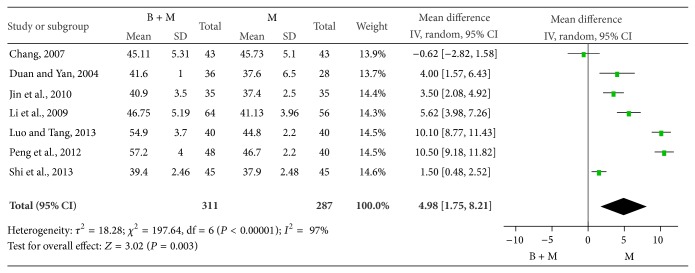
Median MNCV of patients in two groups.

*(2) Median SNCV.* The median SNCV was compared between groups in 7 studies [25, 28, 29, 31, 33, 36, 37]. There were 311 patients treated with breviscapine and mecobalamin and 287 patients treated with mecobalamin alone. There was significant heterogeneity among groups (*P* < 0.0001, *I* ^2^ = 97%), and random effects model was employed for pooled analysis. The pooled WMD was 4.98, 95% CI was 1.75–8.21, *Z* was 3.02 (*P* = 0.003), and the diamond on the right side of the vertical line was complete, suggesting that the median SNCV of patients treated with breviscapine and mecobalamin was significantly higher than that of patients treated with mecobalamin alone (Figure 6). 

**Figure 7:**
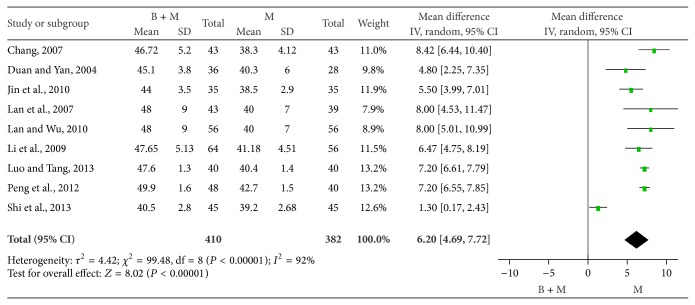
Peroneal MNCV of DPN patients in two groups.

*(3) Peroneal MNCV.* The peroneal MNCV was compared between groups in 9 studies [25, 26, 28, 29, 31, 33, 34, 36, 37]. There were 410 patients treated with breviscapine and mecobalamin and 382 patients treated with mecobalamin alone. There was significant heterogeneity among groups (*P* < 0.00001, *I* ^2^ = 92%), and random effects model was employed for pooled analysis. The pooled OR was 6.20, 95% CI was 4.69–7.72, *Z* was 8.02 (*P* < 0.0001), and the diamond on the right side of the vertical line was complete, suggesting that the peroneal MNCV of patients treated with breviscapine and mecobalamin was significantly higher than that of patients treated with mecobalamin alone (Figure 7). 

**Figure 8:**
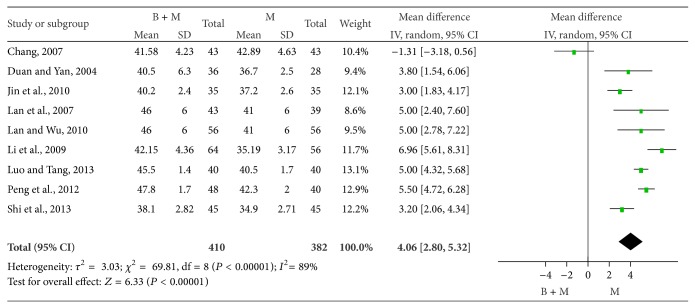
Peroneal SNCV of DPN patients in two groups.

*(4) Peroneal SNCV.* The peroneal SNCV was compared between groups in 9 studies [25, 26, 28, 29, 31, 33, 34, 36, 37]. There were 410 patients treated with breviscapine and mecobalamin and 382 patients treated with mecobalamin alone. There was significant heterogeneity among groups (*P* < 0.00001, *I* ^2^ = 89%), and random effects model was employed for pooled analysis. The pooled OR was 4.06, 95% CI was 2.80–5.32, *Z* was 6.33 (*P* < 0.0001), and the diamond on the right side of the vertical line was complete, suggesting that the peroneal SNCV of DPN patients treated with breviscapine and mecobalamin was significantly higher than that of patients treated with mecobalamin alone (Figure 8).

### 3.4. Adverse Events

After therapy for 2–6 weeks, patients were tolerant to both therapies and there were no severe side effects related to therapies. However, there were mild side effects: 1 with mild headache, 2 with mild nausea [29], 5 with itching [29, 30, 35], and 2 with palpitation at injection [35]. However, these side effects resolved soon after discontinuation of therapy.

### 3.5. Publication Bias

**Figure 9:**
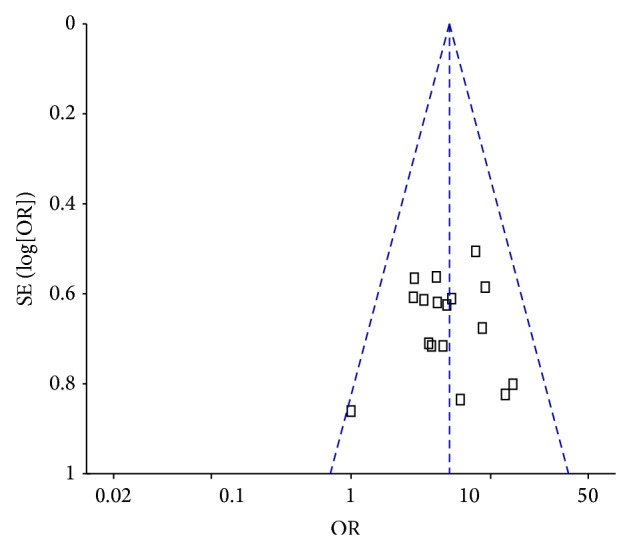
Funnel plot for B-M group versus M alone group for DPN.

Funnel plot was used for the evaluation of publication bias of studies included in this meta-analysis. Results showed that the funnel plot was nearly symmetrical, suggesting no publication bias in these studies (Figure 9).

## 4. Discussion

DM is one of the most common diseases, and its incidence is increasing worldwide with the acceleration of economic developments and the pace of living. It is estimated that the prevalence of DM in 2030 will be 50.7% higher than that in 2011, and about 48% of new DM cases will be found in China and India [43]. DPN is one the most common complications of DM. Dyck et al. [44] found that the incidence of DPN was 66% and 59% in patients with type 1 and type 2 DM, respectively. DPN may cause damage to the motor, sensory, and autonomic nerves and even cause limb gangrene and amputation.

The pathogenesis of DPN is very complicated and is currently regarded as a result of interaction among multiple factors under a hyperglycemic state including suppressions of glycation end products generation, changes in protein kinase C signaling pathway [45], activation of polyol pathway [46], and increases in cytokines due to ischemia and/or hypoxia [47]. Modern pharmacological study [48] showed that breviscapine was able to dilate blood vessels, reduce blood viscosity, inhibit platelet aggregation, increase activities of plasma endothelin, renin, and angiotensin, dilate arterioles, improve microcirculation, increase blood supplies of nerves, improve ischemia and hypoxia, and elevate nerve conduction velocity, which were helpful to improve the symptoms and signs of DPN and increase the sensory nerve conduction velocity of limbs. Mecobalamin is a derivative of coenzyme vitamin B12 and may act to repair myelin to improve DPN. Breviscapine and mecobalamin may exert synergistic effects on DPN in different mechanisms and significantly increase the therapeutic efficacy [49].

Liu [50] found that breviscapine could improve the therapeutic efficacy in DPN patients in a systematic review, but the safety was not good. Our results showed that the therapeutic efficacy of breviscapine in combination with mecobalamin in DPN patients was remarkably superior to that of mecobalamin alone. Among all the studies included in this meta-analysis, the combined therapy group was significantly different from control group. Of these studies, 7 confirmed that breviscapine could improve or even cure the symptoms of motor and sensory nerves in DPN patients [25, 28, 29, 31, 33, 36, 37]. In addition, therapy with breviscapine and mecobalamin had no severe side effects. Hu et al. [51] found that the inner wall of blood vessels of lower limbs was thickened and became rough, and vascular stenosis and atheromatous plaques were observed, suggesting that thrombosis, vascular narrowing, and increased blood viscosity were closely associated with DPN. In clinical practice, DPN is treated with mecobalamin, which is effective in relieving the pain, numbness, and hypoesthesia, but the therapeutic efficacy is still unsatisfactory [52]. Thus, some researchers [25, 26, 28–42] attempted to apply breviscapine in combination with mecobalamin in the therapy of DPN. Results showed that breviscapine was able to significantly increase the therapeutic efficacy of DPN, reduce the blood viscosity, and elevate the MNCV and SNCV, and other effects related to elevated therapeutic efficacy were also superior to those after monotherapy with mecobalamin. Similar findings were also obtained in the present study.

However, meta-analysis still has limitations. In a majority of studies, the method used for randomization was not addressed, whether there was allocation concealment of randomization is unclear, the blinding method was not described in these studies, and whether intention to treat analysis was performed was still unclear. In addition, patients in these studies were not followed up after interventions, and thus the recurrence of DPN after combined therapy with breviscapine and mecobalamin was not able to be determined.

## 5. Conclusion

Meta-analysis shows that combined therapy with breviscapine and mecobalamin is safe and effective for DPN patients. The number of studies included in this meta-analysis is small, there is still heterogeneity in the nerve conduction velocity among studies, and the methodology in these studies has defects. Thus, more high-quality, controlled, randomized clinical trials are needed to further confirm the therapeutic efficacy of breviscapine in combination with mecobalamin in DPN patients.

## Figures and Tables

**Table 1:** Characteristics of included studies.

Trial	Number	Age (Y)	Duration of diabetes (Y)	Duration of DPN (Y)	Treatment drugs/day			Main outcome measures	Course of treatment	Adverse events
					B + M		M			
	(B + M/M)	(B + M/M)	(B + M/M)	(B + M/M)	B + M	M				
Shi et al. (2013) [25]	90 (45/45)	58.4 ± 11.2/57.9 ± 10.8	11.2 ± 2.3/10.7 ± 2.6	3.01 ± 0.93/3.15 ± 0.87	50 mg iv.	1.5 mg n.r.	1.5 mg n.r.	TER, SNCV, MNCV	6 w	0
Lan and Wu (2010) [26]	82 (43/39)	53.2 ± 3.7/53.6 ± 3.6	9.3 ± 8.3/9.54 ± 3.1	4.3 ± 1.3/4.2 ± 1.2	150 mg ivgtt.	0.5 mg im.	0.5 mg im.	TER, SNCV	4 w	n.r.
Chang (2007) [29]	86 (43/43)	69.4/63.7	12.0/11.0	4/3.5	40 mL ivgtt.	0.5 mg po.	0.5 mg po.	TER, SNCV, MNCV	30 d	5/0
Chen and Shao (2008) [30]	132 (68/64)	72 ± 12.3/69 ± 11.9	n.r.	n.r.	30 mL ivgtt.	1.5 mg po.	1.5 mg po.	TER	2 w	2/0
Duan and Yan (2004) [31]	64 (36/28)	55.5 ± 5.2	5.5 ± 4.2/n.r.	3.6 ± 2.5/n.r.	30 mL ivgtt.	0.5 mg im.	0.5 mg im.	TER, SNCV, MNCV	6 w	n.r.
Feng et al. (2009) [32]	91 (46/45)	n.r.	n.r.	n.r.	20 mL ivgtt.	1.0 mg ivgtt.	1.0 mg ivgtt.	TER	4 w	n.r.
Jin et al. (2010) [33]	70 (35/35)	57 ± 10/56 ± 10	8.5 ± 3.4/8.5 ± 3.2	n.r.	20 mL ivgtt.	0.5 mg im.	0.5 mg im.	TER, SNCV, MNCV	4 w	n.r.
Lan et al. (2007) [34]	82 (43/39)	53.2 ± 3.7/53.6 ± 3.6	9.3 ± 8.3/9.54 ± 8.1	4.3 ± 1.3/4.2 ± 1.2	150 mg ivgtt.	0.5 mg im.	0.5 mg im.	TER, SNCV	4 w	n.r.
Li et al. (2009) [28]	120 (64/56)	60 ± 6.52/60.5 ± 8.33	5.5 ± 4.2	2.6 ± 0.4/2.8 ± 0.5	20 mL ivgtt.	0.5 mg im.	0.5 mg im.	TER, SNCV, MNCV	4 w	0
Li et al. (2006) [35]	56 (29/27)	55 ± 3/56 ± 4	6.75 ± 1.2/6.8 ± 1.2	2.15 ± 0.21/1.98 ± 0.23	135 mg ivgtt.	1.5 mg po.	1.5 mg po.	TER	4 w	2/0
Luo and Tang (2013) [36]	80 (40/40)	61.3 ± 12.08/62 ± 11.87	3.02 ± 1.75/3.04 ± 1.93	5.57 ± 2.03/5.42 ± 2.2	70 mg ivgtt.	1.0 mg iv.	1.0 mg iv.	TER, SNCV, MNCV	2 w	n.r.
Peng et al. (2012) [37]	88 (40/48)	61.56 ± 12.18/62.36 ± 6.25	11.83 ± 2.3/12.04 ± 4.52	4.9/5.5	75 mg ivgtt.	1.0 mg ivgtt.	1.0 mg ivgtt.	TER, SNCV, MNCV	2 w	n.r.
Sun (2014) [38]	60 (30/30)	53.2 ± 3.6/51.7 ± 2.8	10.6 ± 4.2/9.5 ± 3.4	n.r.	20 mL ivgtt.	1.0 mg ivgtt.	1.0 mg ivgtt.	TER, SNCV, MNCV	2 w	n.r.
Wang (2013) [39]	68 (34/34)	62.4 ± 8.4	n.r.	n.r.	200 mg ivgtt.	1.5 mg po.	1.5 mg po.	TER	4 w	2.0/3.0
Wu and Zhang (2007) [40]	74 (38/36)	48/47	7.34/7.28	2.32/2.24	40 mL ivgtt.	500 mL im.	500 mL im.	TER	2–6 w	n.r.
Zhang (2012) [41]	60 (30/30)	59.06 ± 7.82	7.15 ± 1.32	2.06 ± 0.59	50 mg ivgtt.	1.5 mg po.	1.5 mg po.	TER	4 w	0
Zhou (2009) [42]	65 (33/32)	n.r.	n.r.	n.r.	40 mL ivgtt.	1.5 mg po.	1.5 mg po.	TER	4 w	0
